# Gut Microbiome and Carotid Artery Intima-Media Thickness: A Narrative Review of the Current Scenario

**DOI:** 10.3390/diagnostics14222463

**Published:** 2024-11-05

**Authors:** Barbara Pala, Giuliano Tocci, Giulia Nardoianni, Emanuele Barbato, Amedeo Amedei

**Affiliations:** 1 Division of Cardiology, Department of Clinical and Molecular Medicine, Sant’Andrea Hospital, University of Rome Sapienza, 00189 Rome, Italy; giuliano.tocci@uniroma1.it (G.T.); giulianardoianni@gmail.com (G.N.); emauele.barbato@uniroma1.it (E.B.); 2 SOD of Interdisciplinary Internal Medicine, Azienda Ospedaliera Universitaria Careggi (AOUC), 50134 Florence, Italy; 3 Network of Immunity in Infection, Malignancy and Autoimmunity (NIIMA), Universal Scientific Education and Research Network (USERN), 50139 Florence, Italy

**Keywords:** gut microbiome, intima-media thickness, carotid atherosclerosis, ultrasound, carotid plaques

## Abstract

Up to the last update, the gut microbiome (GM) had been associated with a different physiologic host process, including those affecting cardiovascular health. The carotid intima-media thickness (IMT) is an indicator of atherosclerosis and cardiovascular risk. The GM influence on atherosclerosis progression has garnered growing attention in recent years but the consensus in subclinical atherosclerosis remains elusive. The aim of this narrative review is to investigate the connection between the GM and carotid IMT, encompassing mechanisms like the microbiome impact on metabolite production, and systemic inflammation, and its effects on endothelial function. The literature analysis revealed that the GM appears to exert an influence on carotid IMT development, likely through mechanisms involving metabolites’ production, systemic inflammation, and endothelial function modulation. Additional research, however, is needed to finely elucidate the relationship between the GM and atherosclerosis. Specifically, more extensive studies are required to pinpoint individuals at the highest risk of developing atherosclerosis based on their GM composition. This will facilitate the enhancement and optimization of cardiovascular disease prevention strategies and enable the treatments’ customization for each patient. Further investigations are required to refine patient outcomes in the context of probiotics and other interventions aimed at improving microbiome composition and function.

## 1. Introduction

Atherosclerosis (AS) is a chronic disease, often asymptomatic, associated with increased risk of major cardiovascular (CV) complications, reduced life expectancy, and poor cardiovascular prognosis, representing a significant global health challenge [1]. AS may occur in both large and small arteries in humans, but typically develops in areas of abnormal or impaired blood flow. Actually, AS disorders, such as acute coronary syndrome and stroke, stand as the foremost contributors to mortality and incapacitation within emerging nations [2].

Hallmark features include the buildup of low-density lipoproteins within arterial walls, activation of endothelial cells, and expression of leukocyte adhesion molecules and chemokines. These processes attract innate and adaptive immune cells (monocytes and T cells) to the arterial intima, triggering a localized inflammatory response [3]. Moreover, AS progression can culminate in local proteolysis, the disruption of AS plaques, and, lastly, thrombus formation, potentially leading to ischemic events such as acute coronary syndromes and myocardial infarction [4]. In light of these complex mechanisms, human AS can be comprehensively viewed as both a metabolic and inflammatory disorder [1].

Among different localizations, carotid AS is linked to a high risk of CV complications, mostly stroke. The thickness of the carotid artery wall, known as the carotid intima-media thickness (IMT), has been extensively studied as a marker of CV risk [5]. The measurement criteria for carotid arteries using ultrasonography are as follows: (1) increased carotid intima-media thickness, defined as a thickness exceeding 0.9 mm; (2) carotid plaque, identified as a localized thickening of the carotid intima-media to 1.2 mm or more, which encroaches into the lumen; and (3) carotid stenosis, diagnosed when there is an obstruction of 50% or greater in the carotid artery’s lumen [6]. It should be noted, however, that beyond the size of the atherosclerotic plaque, its stability plays a crucial role as a major risk factor for stroke. This aspect has gained relevant clinical attention in the management of carotid stenosis conditions. Indeed, vulnerable plaques are characterized by a sizable cholesterol-rich core, low levels of calcium, and thin fibrous caps—all conditions able to increase the risk of stroke. Consequently, it is imperative to explore effective methods to enhance the stability of carotid atherosclerotic plaques [2].

The significance of the gut microbiome (GM) in human well-being and its complex connections with vascular conditions, encompassing cardiovascular disease (CVD) and chronic kidney disease (CKD), have garnered heightened recognition in recent years [7]. Recent findings suggested that the GM could potentially exert a relevant influence on host vascular physiology, promoting AS development and progression and, thus, favoring CVD onset [8]. However, it is relevant to note that a consensus on this matter has yet to be reached. Studies have reported the involvement of several bacterial products in AS development, such as immune activators and diet-related metabolites, especially the emerging trimethylamine N-oxide (TMAO), which holds a pivotal role in this context [9,10]. While the connection between specific bacterial groups and AS has been established, numerous inquiries persist, leaving us with a partial understanding of the potential role of the GM in the development of atherosclerosis and CVD [5]. Thus, the growing interest in GM-targeted interventions for both CVD prevention and treatment is justified by numerous microbial pathways associated with CVD risk, notably showing their relative independence from dietary factors and inflammation [9].

In the Moscow Study [11], different metabolic alterations were linked to the varying prevalence of genera in cases involving CV risk factors; for instance, disturbances in glucose metabolism were correlated with higher Blautia and Serratia levels, and arterial hypertension was associated with high Blautia levels. Additionally, the carotid IMT was greater in the group with a lower microbial diversity.

In a Swedish study [12], patients with symptomatic AS displayed an enrichment of the genus Collinsella, while Roseburia and Eubacterium were more abundant in healthy controls (HCs).

Moreover, patients with AS exhibited increased levels of Enterobacteriaceae and *Streptococcus* spp. [13].

**Figure 1:**
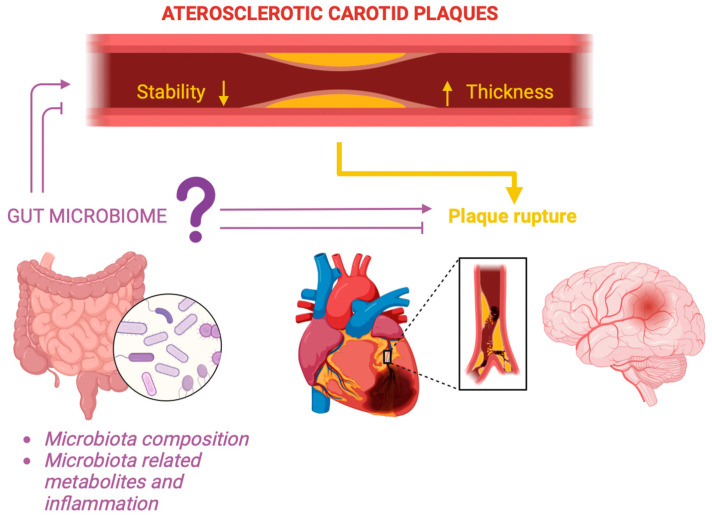
The thickness of the carotid artery wall and its stability plays a crucial role as a major risk factor for strokes, directly, and acute myocardial infarction, indirectly. How does the GM alter the atherosclerotic plaques scenario, and what does it alter in it? Created with Biorender.com.

Our narrative review aims to explore potential associations between the GM diversity and carotid IMT, by summarizing the documented evidence (Figure 1).

We focused on carotid plaques because they are considered important predictors of atherosclerotic disease and can be easily evaluated using non-invasive and repeatable Doppler ultrasound methods. Summarizing the existing literature evidence, we can provide significant clinical insights potentially generalizable to atherosclerosis and atherogenesis. Furthermore, this approach can offer valuable directions for easily implementable and non-invasive observational or interventional clinical studies.

Considering that lifestyle influences the GM [14], confirming the link between the microbiota and cIMT in this review could be one way in which a healthy lifestyle helps maintain a low cIMT, complementing a healthy, cholesterol-lowering diet.

The motivational questions were primarily “Is the gut microbiome composition linked with AS? Are there specific species more prevalent in patients with increased intima-media thickness compared to healthy controls?”; moreover, “Does the carotid IMT increase or decrease?”. Additionally, “How does the GM alter the AS scenario, and what does it alter in it? Does the gut microbiome act directly or indirectly? Are there particular bacterial metabolites that are predominantly implicated?”.

The carotid intima-media thickness (cIMT) and plaque represent distinct yet interrelated cardiovascular phenomena. The cIMT is visualized as a double-line pattern on an ultrasound, reflecting the intima–lumen and media–adventitia boundaries of the carotid artery. According to these ESC guidelines [15], a carotid IMT greater than 0.9 mm has been reaffirmed as a marker of asymptomatic organ damage and is consistently associated with increased cardiovascular risk [16]. In contrast, plaques are focal lesions that protrude into the arterial lumen, typically defined as having a thickness of at least 0.5 mm or encroaching upon 50% of the surrounding cIMT, with a critical thickness measurement exceeding 1.5 mm. It is important and relevant to note that the progression of the cIMT can lead to plaque formation if preventive measures are not implemented. This relationship underscores the significance of monitoring the cIMT as a potential precursor to more advanced atherosclerotic changes [17,18,19].

### 1.1. Gut Microbial Composition in CVD

In addition to viruses, fungi, and archaea, the human GM primarily consists of two prominent bacterial phyla, namely, Firmicutes and Bacteroidetes, which collectively make up over 90% of the overall microbial community. Additionally, there are other less dominant phyla, including Proteobacteria, Actinobacteria, and Verrucomicrobia [20,21].

As the predominant bacteria in the gut, Bacteroides and Firmicutes are generally resilient to acute disturbances. However, changes in their populations are frequently linked to specific pathological conditions in the human body [20]. Notably, this composition tends to remain relatively stable even in the presence of acute disruptions, as its adaptability enables it to swiftly revert to its original state [22]. Emoto and colleagues observed a significant increase in the abundance of Firmicutes and a corresponding decrease in Bacteroides within the gut microbiota of patients with coronary artery disease (CAD) [23].

In recent years, the GM has been implicated in the development of atherosclerotic plaques, as bacterial DNA has been detected in arterial plaques in humans [24]. Indeed, focusing on the presence or absence of carotid AS several studies, performed in different countries, tested the potential associations between specific gut bacteria and carotid plaques [5,8,12,25].

Szabo and colleagues [5] observed that the most prevalent bacterial phyla (included Firmicutes, Bacteroidetes, Proteobacteria, Actinobacteria, and Verrucomicrobia, with the exception of Chloroflexi) were consistent in patients with an elevated IMT than in the control group. Of note, they firstly reported an increased Firmicutes-to-Bacteroidetes ratio in the increased carotid IMT group. Wang and colleagues [8] investigated the potential links between the diversity and GM composition and the presence of carotid AS. They found significant differences in the relative abundance of certain bacteria; specifically, higher levels of Fusobacterium and Proteus were associated with an increased likelihood of carotid artery plaque, while higher levels of Odoribacter were linked to a decreased likelihood of plaque occurrence.

Moreover, Zhu and colleagues [25] reported associations between several gut bacterial genera (such as Faecalicatena and Libanicoccus) and subclinical AS (assessed as carotid IMT measurements) in a population-based cohort study of elderly Chinese individuals. Finally, a Swedish study [12], involving 25 participants, revealed that Collinsella was more abundant in patients with symptomatic carotid AS, whereas Roseburia and Eubacterium were enriched in the control group.

In the broader CVD context, several prior studies have explored the connections between GM and atherosclerosis or CVD in humans, yielding different findings: a case–control investigation in Shanghai [26], revealed a lower GM α-diversity in 70 patients with coronary artery disease compared to 98 HCs. A relatively larger case–control study among Chinese [13] did not identify significant disparities in GM α-diversity between 218 patients with atherosclerotic cardiovascular disease and 187 HCs. Furthermore, these case–control studies have reported alterations in various bacterial taxa within CVD groups, but only a few outcomes exhibit consistency across studies, such as the reduced presence of Roseburia genus in CVD patients [13,26].

The GM not only affects the formation and thickness of plaques; it also impacts their vulnerability or stability. Some researchers have observed that DNA from gut bacteria can activate macrophages through Toll-like receptor 2 (TLR2) and TLR4, thereby triggering the immune system and impairing plaque stability [27].

Intravascular ultrasound (IVUS) and optical coherence tomography (OCT) used to evaluate the nature of plaques have identified particular species as being associated with the presence of atherosclerosis in thin-cap fibroatheromas. Additionally, studies have shown that these two bacteria are also linked to larger plaques in blood vessels [28]. Akkermansia has been shown to reduce plasma lipopolysaccharide (LPS) levels in obese patients with metabolic syndrome. This decrease is associated with a reduced promotion of atherosclerosis and the formation of vulnerable plaques [29]. Indeed, LPS levels were found to be significantly higher in individuals with carotid plaques compared to those without, suggesting that bacteria associated with elevated LPS production may have a positive correlation with atherosclerosis [27].

### 1.2. Microbiota-Related Metabolites in CVD

Microbiota-related metabolites have been proposed as potential intermediaries linking the GM with CVD [8]. It has been reported that the GM metabolites, specifically TMAO and LPSs, can cause vascular endothelial damage, leading to impaired plaque stability and promoting thrombosis. In particular, LPSs can destabilize plaques by promoting a systemic inflammatory response and activating inflammatory cells. Conversely, some short-chain fatty acids (SCFAs), a peculiar bi-product of bacterial fermentation in the large intestine from undigested food, contribute to the stabilization of coronary atherosclerotic plaques [27].

TMAO is a metabolite produced by the liver, primarily through the action of flavin monooxygenase 3 (FMO3), after being metabolized by intestinal microorganisms from dietary nutrients commonly found in a Western diet (e.g., lecithin, choline, and carnitine). This metabolite is ultimately secreted by the liver following its formation through oxidation [30]. Recent studies have increasingly highlighted the elevated levels of plasma TMAO in atherosclerosis, suggesting that it may can speed up atherosclerosis progression. Therefore, decreasing TMAO levels is considered a promising approach to preventing atherosclerosis. For instance, one study documented that TMAO administration to apoE−/− mice [31] resulted in a significant increase in total plaque area, along with elevated levels of triglycerides, total cholesterol, and low-density lipoprotein cholesterol. Moreover, TMAO’s role in the stability of carotid atherosclerotic plaques was investigated. Shi and colleagues [2] demonstrated that a reduction in TMAO levels significantly decreased carotid plaque size and enhanced plaque stability. Regarding the mechanisms involved, the TMAO-induced instability in carotid atherosclerotic plaques might occur through the inhibition of macrophage polarization on the M2 subset and efferocytosis (the process by which macrophages remove dead cells). In support of this, a prospective study conducted on HIV-infected patients [32] revealed a significant association between elevated TMAO plasma levels and an increased risk of carotid artery plaque formation.

LPSs can translocate from the intestinal lumen to the bloodstream through the intestinal mucosa when the intestinal barrier permeability increases. This translocation contributes to atherosclerosis development. Carnevale and colleagues found that macrophages, which, as previously mentioned, play a crucial role in atherosclerotic formation and plaque stability, were present in larger volumes in LPS- and TLR4-positive carotid plaque profiles compared to LPS- and TLR4-negative carotid plaque profiles [33].

### 1.3. Microbiota and Systemic Inflammation: Modulation of Endothelial Function

As well-documented, atherosclerosis is a complex and multifactorial disease involving various cell types; the process of plaque development begins with endothelial dysfunction, which can result from factors like hypertension, diabetes, obesity, and elevated cholesterol levels. This dysfunction leads to the buildup of low-density lipoprotein (LDL) cholesterol between the layers of the arterial wall. Within atherosclerotic lesions, macrophages play an active role in engulfing and accumulating lipoproteins, leading to the development of foam cells containing lipid droplets, and, thus, it is a key contributor to lipid storage and the subsequent growth of atherosclerotic plaques [34]. In plaque lesions, there are two distinct macrophage phenotypes: the proinflammatory (M1) and the anti-inflammatory (M2) phenotypes. As previously reported, these macrophage phenotypes are dynamic and can switch between each other when exposed to various stimuli, including lipoproteins, lipids, cytokines, chemokines, and bioactive molecules [2].

Indeed, the activation of M1 macrophages by factors like LPSs and interferon-γ (IFN-γ) leads to an increased expression of proinflammatory mediators such as TNF-a, iNOS, and IL-6 (interleukin-6), which are crucial in plaque vulnerability. Conversely, the activation of M2 macrophages, promoted by cytokines like IL-4 and IL-13, contributes to tissue repair and the clearance of cellular debris [2]. The activation of TLR2, which is notably expressed by endothelial cells within atherosclerotic plaques, plays a role in promoting endothelial apoptosis and denudation. TLR2 ligands encompass certain components found in Gram+ bacteria, suggesting that infectious factors could potentially contribute to atherothrombosis through this mechanism. In other words, these findings suggest that infectious agents may have a partial involvement in both the development of atherosclerosis and CV onset [1].

### 1.4. Oral Microbiota Impact on Atherosclerotic Plaque

As previously argued, the presence of bacteria within atherosclerotic plaque samples has been confirmed, hinting at their potential involvement in the progression of cardiovascular disease. Notably, the genus Curvibacter was particularly predominant across all plaque samples [1]. In addition, periodontal disease has been linked to cardiovascular and cerebrovascular events, especially with inflammation in the periodontium believed to elevate the host systemic inflammatory tone, potentially impacting plaque composition and rupture.

Previous studies have documented in atherosclerotic plaques the presence of bacteria usually resident in both the oral cavity and the gut. Additionally, research by Hyvärinen and colleagues [35] found an increase in the periodontal pathogen *Aggregatibacter actinomycetemcomitans* in the saliva of patients with coronary disease (whether symptomatic or asymptomatic) compared to HCs. Furthermore, Fak and colleagues [36] suggested a potential association between the abundance of Anaeroglobus in the oral cavity and symptomatic atherosclerosis. Finally, a recent study documented the presence of infiltrating T lymphocytes and tissue-associated microbiota in freshly collected samples of calcified aortic valve [37]. However, the existence of a definitive causal link between periodontitis and atherosclerosis remains inconclusive. Indeed, Isoshima and colleagues [34] revealed distinct microbiome patterns in atheromatous plaques in the presence or absence of periodontitis, suggesting that oral bacteria do not have a direct impact on the development of atheromatous plaques.

## 2. Discussion

It is well-established that GM plays a significant role in influencing the development of both cardiovascular and cardiometabolic diseases; indeed, we reported and discussed the associations between both oral bacteria and the GM and the development of atherosclerotic plaques.

Microbial diversity and specific bacterial species may contribute to cardiovascular risk through their effects on lipid metabolism, immune modulation, and inflammatory responses. By addressing these broader mechanisms, we aim to provide a more holistic view of how the GM may impact cardiovascular health.

Confirming the link between the GM and subclinical carotid atherosclerosis establishes the gut as a viable target for probiotic and dietary interventions aimed at restoring a healthy microbiota. This approach may help reduce chronic inflammation, potentially preventing or even reversing atherosclerosis and lowering the risk of major cardiovascular events. Additionally, we emphasize that GM evaluation could become an integral part of cardiovascular risk assessment, serving as an early warning system for patients not yet receiving cardiovascular care. This could facilitate earlier medical intervention and ultimately decrease the incidence of severe cardiovascular outcomes.

Our narrative review of more recent and relevant studies reveals a clear association between alterations in the Firmicutes-to-Bacteroidetes ratio and an increased carotid IMT which may be considered an atherosclerosis marker. This finding is consistent with previous studies, documenting in obese patients a decreased relative abundance of Bacteroidetes compared to normal-weight subjects. Therefore, changes in bacterial composition are generally linked to alterations in the metabolic profile of the microbiota, and the Firmicutes-to-Bacteroidetes ratio may serve as a potential obesity indicator. Interestingly, Szabo and colleagues [5] observed an elevated Firmicutes-to-Bacteroidetes ratio in association with an increased IMT independently of the body mass index (BMI), further emphasizing its role as a marker indicative of the atherosclerotic phenotype [5]. Regarding which specific bacterial species are most strongly correlated with an increased carotid IMT, currently, there are no definitive data available, as different studies have highlighted different species; therefore, we cannot definitively establish bacterial communities as the direct cause of atherosclerosis. Remarkably, more bacteria have been discovered within human carotid artery plaque tissues, although there remains uncertainty about whether these bacterial factors/metabolites play a role, even if partial, in atherosclerosis pathogenesis [38]. In addition, antibiotic administration to many atherosclerosis patients, without resulting in improved clinical outcomes, may have led to unwarranted skepticism regarding bacteria involvement in the development of atherosclerosis [1].

Furthermore, it is not clear which is the source of the plaque infiltrating bacteria. It is possible that oral bacteria can travel through the bloodstream [39]. However, some studies raised doubts about whether these bacteria might merely be non-dispersing microbial contaminants [1], since it has been observed that oral bacteria do not exert a direct influence on the formation of atheromatous plaques [34]. However, it is crucial to emphasize that both commensal (harmless) and pathogenic (disease-causing) microorganisms show the ability to generate biofilm formations adhering to various surfaces. Therefore, apart from the established factors in atherosclerosis development, such as the accumulation of cholesterol and the infiltration of macrophages into the arterial wall, these bacterial biofilm structures may have an additional role in perpetuating the inflammation associated with atherosclerosis [1]. From our point of view, it is plausible that microbial metabolites and/or host inflammatory factors may indirectly impact the composition and the vulnerability of atheromatous plaques.

We highlight the potential influence of GM metabolites such as TMAO and LPSs on different aspects of cardiovascular health. These metabolites have been implicated not only in plaque formation and stability but also in systemic inflammation and endothelial dysfunction, which are critical processes in the development of atherosclerosis and other cardiovascular diseases.

**Figure 2:**
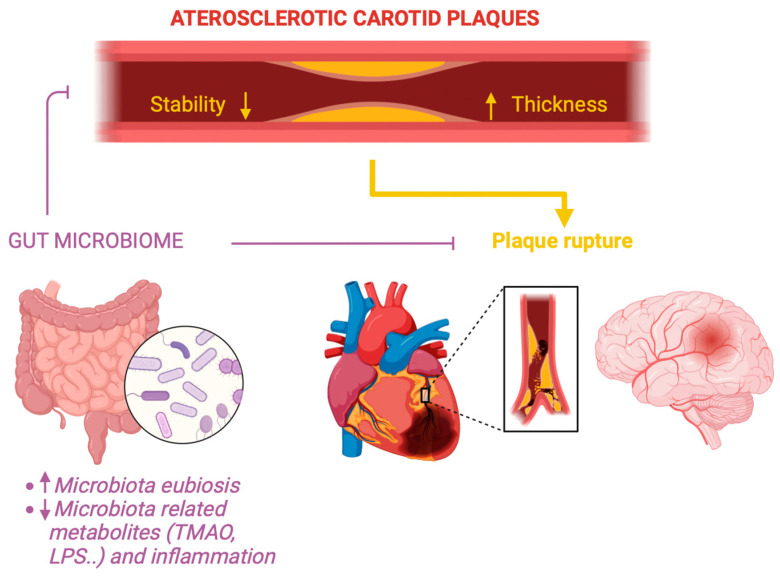
The gut microbiome plays a crucial role in the thickness and stability of the carotid artery wall, directly affecting the risk of stroke and indirectly influencing the risk of acute myocardial infarction. Maintaining correct microbiota eubiosis and a balance of its metabolites is essential for preventing acute cardiovascular syndromes. Created with Biorender.com.

Furthermore, the mechanisms leading to endothelial erosion, another form of arterial surface damage, have remained unclear; yet, recent research suggests the involvement of innate immunity [1]. The activation of TLR2, expressed by endothelial cells, overlying atherosclerotic plaques contributes to endothelial apoptosis and denudation. TLR2 ligands encompass some components from bacterial pathogens, implying that both infectious and endogenous factors might contribute to atherothrombosis through this mechanism [1]. Moreover, given that the GM plays a recognized role in shaping the host innate immune response, its presence or absence, as well as the composition of this densely populated microbial ecosystem, has been connected to atherosclerosis and arterial thrombosis [39]. Finally, as reported, TMAO plays a role in IMT thickening and plaque instability by promoting proinflammatory activity while inhibiting M2 and efferocytosis processes (Figure 2). Supplementary Material summarizes the content of bacterial species and their cardiovascular implications.

### Limitations

In discussing the cIMT as a surrogate marker for atherosclerosis, it is important and crucial to recognize its delicate nature. cIMT values are often expressed in sub-millimetric ranges, and even minor differences can lead to significant variations in patient categorization. This sensitivity raises concerns, especially since measurement protocols can vary and may not adequately account for the asymmetric presentation of atherosclerosis.

Moreover, the lack of high-precision measurement techniques can contribute to variability, complicating the interpretation of cIMT results. These factors underscore the challenges faced in achieving standardized assessments across different studies and trials.

Historically, IMT values have been manually collected by examiners and then averaged. In some cases, post-IMT analysis has involved the retrospective evaluation of raw image data. However, there are now more refined and automated measurement options available to standardize the results. It is worth noting that the utilized experimental approaches in the reviewed articles were not consistently uniform. An additional limitation includes small-sample-size studies and a limited number of prospective studies, as this is a relatively new area of research.

Given these complexities, we opted for a narrative review approach rather than a systematic one, acknowledging the inherent difficulties in conducting comprehensive comparisons among existing studies. By focusing on these nuances, we aim to provide a more holistic understanding of the relationship between the GM and cIMT, while recognizing the limitations of current methodologies in the field.

## 3. Conclusions

In this narrative review, we have endeavored to provide a comprehensive summary of the current knowledge regarding the potential links between the GM and the significant subclinical marker, the carotid IMT. Our analysis of the literature revealed that the GM appears to influence the development of the carotid IMT, likely through mechanisms involving the production of metabolites, the modulation of systemic inflammation, and endothelial function. In summary, our review highlights a significant association between an increased Firmicutes-to-Bacteroidetes ratio and an elevated carotid IMT, which is a recognized marker of atherosclerosis. Alterations in the abundance of these bacteria are frequently linked to cardiovascular conditions, especially coronary artery disease. Various studies have identified different bacterial species correlated with an increased carotid IMT; moreover, the presence of oral bacteria within atherosclerotic plaques suggests their potential role in cardiovascular disease progression. However, definitive data establishing the fine causative mechanism remain elusive. Certain species have been implicated in both the formation and stability of atherosclerotic plaques, suggesting that microbial metabolites such as TMAO and LPSs may indirectly influence plaque composition and vulnerability. Moreover, the interplay between the GM, systemic inflammation, and endothelial dysfunction is complex and significant in atherosclerosis progression, as the activation of immune pathways by microbial factors can influence both plaque development and stability.

To advance our understanding, future research should aim to identify individuals at the highest risk of atherosclerosis based on their GM composition, leading to the optimization of CVD prevention strategies tailored to each patient. Our focus on carotid plaques stems from their role as key indicators of atherosclerotic disease, which can be effectively assessed through non-invasive and repeatable Doppler ultrasound techniques. Moreover, this strategy offers significant potential in guiding the development of straightforward, non-invasive observational or interventional clinical studies, enhancing their feasibility and clinical relevance. Likewise, additional investigations are critical to better refining patient outcomes, especially in the context of probiotics and other interventions designed to enhance microbiome eubiosis.

## Supplementary Materials

The following supporting information can be downloaded at: https://www.mdpi.com/article/10.3390/diagnostics14222463/s1.
