# Supplementary material for: Gut Microbiome and Carotid Artery Intima-Media Thickness: A Narrative Review of the Current Scenario
Source: Diagnostics (Basel). 2024 Nov 5;14(22):2463. doi: 10.3390/diagnostics14222463 (PMC11592993; doi:10.3390/diagnostics14222463)
Supplement: Supplementary file 1 [file diagnostics-14-02463-s001.zip › diagnostics-3265978-supplementary.pdf]

Bacterial Phyla/Species	Implications in Carotid Aterosclerosis
Firmicutes	Increased abundance linked to coronary artery disease (CAD) and higher Firmicutes/Bacteroidetes ratio associated with elevated cIMT.
Bacteroidetes	Decreased levels observed in patients with CAD; stability in gut microbiota composition.
Proteobacteria	Higher levels associated with increased likelihood of carotid artery plaque.
Actinobacteria	Associated with various CVD conditions but specifics vary by study.
Verrucomicrobia	Presence noted in different studies; specific cardiovascular implications less clear.
Fusobacterium	Higher levels linked to an increased risk of carotid artery plaques.
Odoribacter	Associated with a decreased likelihood of plaque occurrence.
Collinsella	More abundant in patients with symptomatic carotid atherosclerosis.
Roseburia	Reduced presence in CVD patients; implicated in maintaining cardiovascular health.
Akkermansia	Shown to reduce plasma LPS levels, associated with decreased promotion of atherosclerosis.
